# Features of Undiagnosed Abdominal Pain and Diagnostic Status of Acute Hepatic Porphyria in Japan: A Retrospective Study

**DOI:** 10.7150/ijms.107826

**Published:** 2025-06-20

**Authors:** Masaki Tago, Yosuke Sasaki, Naoko E. Katsuki, Risa Hirata, Hidetoshi Aihara, Fumiya Komatsu, Kazunobu Une, Taiju Miyagami, Yudai Suzuki, Ren Kawamura, Hiroaki Takeoka, Yuka Yasuoka, Taro Shimizu, Shigeki Nabeshima, Toshio Naito, Susumu Tazuma

**Affiliations:** 1 Department of General Medicine, Saga University Hospital, Saga, Japan.; 2 Department of General Medicine and Emergency Care, Toho University School of Medicine, Tokyo, Japan.; 3 Department of Critical Care and General Medicine, JA Onomichi General Hospital, Hiroshima, Japan.; 4 Department of General Medicine, Juntendo University Faculty of Medicine, Tokyo, Japan.; 5 Department of Diagnostic and Generalist Medicine, Dokkyo Medical University, Tochigi, Japan.; 6 General Medicine, Fukuoka University Hospital, Fukuoka, Japan.; 7 Medical Affairs, Alnylam Japan K.K., Tokyo, Japan.; 8 JR Hiroshima Hospital, Hiroshima, Japan.

**Keywords:** Acute hepatic porphyria, Non-specific abdominal pain, Diagnosis, Retrospective observational study, Urinary markers

## Abstract

**Objective:** The prevalence of acute hepatic porphyria (AHP) in Japan is unknown. To diagnose AHP, identifying populations with a high prevalence of AHP is essential. We focused on non-specific abdominal pain (NSAP); however, the criteria for NSAP vary across studies. Therefore, this study aimed to investigate the diagnostic process of undiagnosed abdominal pain in general medicine clinical practice before proposing a definition of NSAP. In addition, we aimed to examine the potential AHP-related symptoms and implementation of AHP testing in these patients. AHP is a rare but fatal and treatable disease; hence, its early diagnosis is essential.

**Design:** This retrospective observational study was conducted in the general medicine departments of six medical institutions in Japan over a 3-year period beginning on April 1, 2019.

**Participants:** Patients with abdominal pain who underwent abdominal imaging examinations were included.

**Main outcome measures:** The primary outcome was to characterize patients with undiagnosed abdominal pain. In addition, this study aimed to identify situations where physicians attempt to diagnose AHP in patients with abdominal pain.

**Results:** Of the 1915 eligible participants, 317 (16.6%) had undiagnosed abdominal pain, and none of them were diagnosed with AHP in diagnosed abdominal pain. The median patient age was 55 years, and 134 patients were male. Multivariate logistic analysis revealed that hospitalization, dull pain, and the absence of depressive symptoms were associated with abdominal pain. All patients with undiagnosed abdominal pain demonstrated two to four indicative symptoms of AHP. However, none underwent urinalysis for a definitive diagnosis of AHP.

**Conclusions:** Depressive symptoms and the absence of dull pain were associated with undiagnosed abdominal pain. Hospitalization for examination contributed to improving the diagnosis of abdominal pain. Despite the presence of indicative symptoms, urinary markers for AHP diagnosis were not measured. Establishing a diagnostic strategy for undiagnosed abdominal pain would provide better opportunities for patients with NSAP and could help shorten the diagnostic journey for those with rare diseases such as AHP.

## Introduction

Many patients present with abdominal pain (AP), including acute abdomen (5-10%), as the primary complaint in general practice and emergency departments 1. Criteria for non-specific abdominal pain (NSAP) vary across studies. Some treat NSAP as synonymous with undiagnosed AP or AP of unknown origin 2,3; some require unnecessary surgery, hospitalization, laboratory test results, or imaging findings 4,5; and some set the duration of AP 4. Therefore, the population with NSAP is suspected to vary across studies. In particular, the inclusion of computed tomography (CT) in imaging tests is an important consideration in the NSAP criteria. Reportedly, 30% of the final diagnoses of AP differed from the initial diagnosis before standard evaluation with ultrasonography and/or CT 2, 19% of AP diagnoses changed after CT from undiagnosed to diagnosed or from diagnosed to undiagnosed AP, and the distribution of diagnoses included in diagnosed AP cases changed from that in the past decade following the widespread use of CT.^5^ Given Japan's high penetration and frequency of CT use 6,7, the populations with NSAP may also differ across countries.

Patients with acute abdomen included undiagnosed abdominal pain 8, and NSAP that develops in acute abdomen may include rare diseases such as acute hepatic porphyria (AHP). Therefore, the criteria used for NSAP and what is included in the differential diagnosis are of great importance for diagnosing rare diseases, particularly those for which treatments are available and necessary. AHP is a rare, hereditary, but treatable disease that manifests with acute attacks of severe AP and indeterminate complaints that interfere with daily activities and quality of life 9. Some patients can experience chronic manifestations along with potentially long-term complications, including chronic kidney disease and liver cell carcinoma 10, and on rare occasions, they can experience respiratory failure and paralysis during an acute attack 11. However, AHP diagnosis requires non-routine examinations based on clinical signs, including urinalysis findings for 5-aminolevulinic acid (ALA) and porphobilinogen (PBG), genetic abnormalities, and organic cause exclusion, which contribute to a diagnostic delay of up to 15 years from symptom onset 12. Therefore, AHP should be considered in the evaluation of all patients with recurrent severe abdominal pain that is not attributable to common causes 13. In Japan, no algorithm dedicated to the close examination of NSAP exists. Hence, for diagnosing AHP, establishing a definition of NSAP is essential, and this study aimed to substantially contribute to this effort. Definitive diagnostic criteria for identifying patients with NSAP among those with undiagnosed AP remain unavailable, and specific procedures are crucial for diagnosing such diseases. Diagnosis would be facilitated if testing could be performed. However, the cost-effectiveness of testing cannot be measured because AHP frequency in Japan is unknown. It is only known that patients with AHP exist in Japan 10.

Therefore, this study aimed to characterize undiagnosed AP and examine potential AHP-related symptoms using undiagnosed AP and AHP testing to determine AHP frequency in patients with AP.

## Methods

### Ethics Statements

This study complied with the Declaration of Helsinki, Ethical Guidelines for Life Sciences and Medical Research Involving Human Subjects 14, and the Act on the Protection of Personal Information 15. The study protocol was approved by the Institutional Review Board for Ethics of Saga University Hospital (approval number: 2022-09-SC-05). Information about the study was disclosed, and patients who refused to participate were excluded. This study was designed and conducted in accordance with the Strengthening the Reporting of Observational Studies in Epidemiology Statement 16.

### Study Design and Population

This retrospective medical record survey investigated the diagnostic status of patients with AP based on electronic medical records from six Japanese regional medical institutions accredited by the Japanese Society of Hospital General Medicine (JSHGM). Accredited institutions must meet two criteria: having a general outpatient clinic or general hospital beds and employing at least one JSHGM-accredited general physician. JSHGM was established in 2010 and currently has approximately 2,200 members 17.

Eligible participants included patients with AP who underwent abdominal imaging at one of six JSHGM-accredited regional medical institutions in Japan (Table S1). The inclusion criteria were patients presenting with AP who visited the general medicine department and had an order for imaging examinations, including abdominal CT, upper or lower gastrointestinal endoscopy, or abdominal ultrasonography in their medical records, between April 1, 2019, and March 31, 2022, irrespective of age and sex.

### Data Collection

Two research physicians from each medical institution (Table S1) independently reviewed the medical records of eligible patients to identify diagnoses. Discrepancies were discussed, and the final diagnosis was determined by consensus. Information was obtained from data closest to the date of the first AP visit. A data management company (Nouvelle Place Inc., Tokyo, Japan) anonymized and integrated the data from each medical institution, which were then used for analysis. Patients with undiagnosed AP were defined as those without a final diagnosis or those coded as R10 according to the International Classification of Diseases 18, indicating AP or pelvic pain with symptoms, signs, and abnormal clinical and laboratory findings not classified elsewhere 18. The remaining patients were categorized as having been diagnosed with AP.

### Statistical Analysis

Table S2 lists the univariate logistic regression analysis conducted using the parameters as explanatory variables and the presence of a diagnosis as the objective variable. AHP-related symptoms were cited from the clinical findings of the diagnostic criteria for AHP in Japan (Table S3). Multivariate logistic analysis was performed using clinically significant parameters from the univariate logistic regression analysis as explanatory variables, with undiagnosed or diagnosed AP as the objective variable. Pearson product-moment and Spearman rank correlation coefficients were calculated for each combination of explanatory variables in the multivariate logistic analysis to confirm collinearity. Combinations with correlation coefficients >0.7 and *p*-values <0.05 were not simultaneously used as explanatory variables in the multivariate logistic analysis.

Summary statistics are present for continuous variables, and the number of cases and percentages are expressed for nominal variables. The student *t*-test was used to analyze continuous variables, whereas the Fisher exact test was used to analyze nominal variables. The diagnostic status of AHP was investigated in the undiagnosed group based on diagnostic criteria (Table S3). In principle, the missing values are not complemented. The statistical analysis software R (version 4.2.2; R Foundation for Statistical Computing, Vienna, Austria) was used for all statistical analyses, and the significance level was set to 5% (two-sided).

## Results

**Figure 1:**
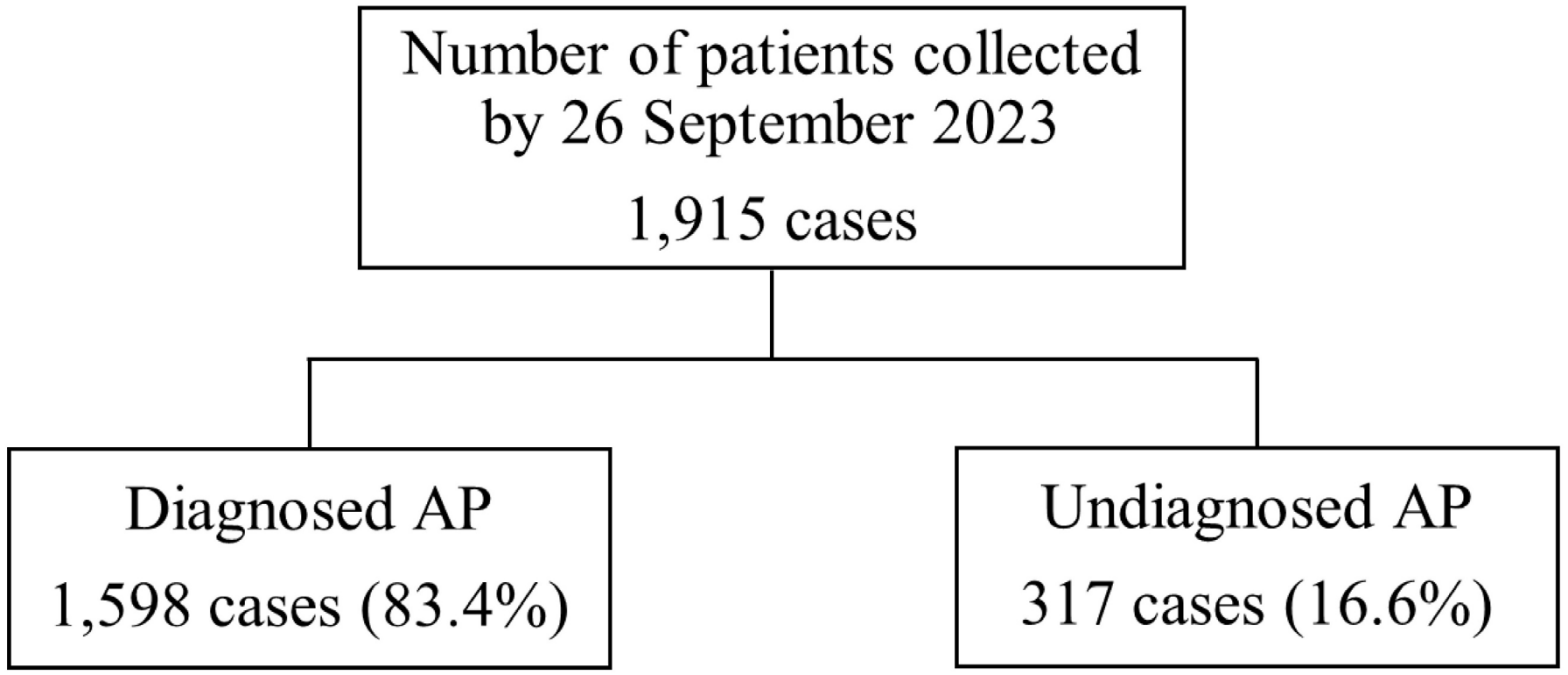
Flow chart for classifying undiagnosed and diagnosed abdominal pain

Of the 1915 patients, 1598 (83.4%) and 317 (16.6%) were included in the diagnosed and undiagnosed groups, respectively (Figure 1). In the undiagnosed group, the median age was 55 years, 42.3% were male, and the body mass indexes were 23.0 and 22.0 kg/m^2^ in males and females, respectively (Table 1). These baseline characteristics were similar between the diagnosed and undiagnosed groups; however, differences were observed in out-of-hour visits, history of being transported by ambulance, number of visits due to AP, and onset time of AP symptoms (Table 1).

The most common diagnoses (>5%) in descending order were infectious enteritis, ureterolithiasis, acute appendicitis, and ileus or bowel obstruction (excluding those associated with malignancy) (Table S4). No patient with AHP was identified.

The rates of abdominal imaging in the undiagnosed group were 92.7%, 35.3%, 30.6%, 15.8%, 13.6%, and 4.1% for abdominal CT, plain abdominal radiography, abdominal ultrasonography, upper endoscopy, lower endoscopy, and abdominal magnetic resonance imaging (MRI), respectively (Table S5). The imaging status was similar between the diagnosed and undiagnosed groups, except for plain abdominal radiography and abdominal MRI.

Univariate logistic regression analysis was performed using the parameters of examination status (examined or not examined; Table S6), AP symptoms (presence or absence; Table S7), associated findings other than AP (presence or absence; Table S8), and other characteristics (Table S9) as explanatory variables, and undiagnosed or diagnosed AP as the outcome. Table 2 summarizes the parameters with odds ratios >1 (*p*<0.05) and <1 (*p*<0.05), with “no parameter” as the reference. The results indicated the following main explanatory variables with an odds ratio of >1.5: hospitalization for examination, blood test result for magnesium, opioid use for pain, examination with plain abdominal radiography, examination with abdominal MRI scan, blood test result for the albumin-globulin ratio, blood test result for plasma osmolality, dull pain, absence of exacerbating factors, non-opioid use for pain, and blood test result for total protein. The main explanatory variables with an odds ratio <0.5 were other exacerbating factors and anxiety symptoms. Multivariate logistic analysis of the explanatory factors listed in Table S10 revealed that hospitalization for examination and dull pain were contributing factors to AP diagnosis, and depressive symptoms were associated with undiagnosed AP (Table 3).

The percentages of patients without hospitalization for examination and those with hospitalization for examination for each examination were 33.8% and 78.0% for electrocardiography (ECG), 51.5% and 66.4% for urinalysis, 27.9% and 39.8% for abdominal ultrasonography, 12.7% and 19.9% for upper endoscopy, 10.2% and 17.6% for lower endoscopy, 35.9% and 71.9% for plain abdominal radiography, 90.7% and 99.1% for abdominal CT, 3.8% and 12.4% for abdominal MRI, 2.7% and 3.5% for transvaginal ultrasonography, 93.6% and 99.6% for blood test results, and 38.2% and 70.6% for blood gas, respectively (Table S11). The diagnoses in >5% of patients included ileus or bowel obstruction (excluding those associated with malignancy) (10.3%), acute appendicitis (10.0%), infectious enteritis (8.3%), acute cholangitis (6.8%), undiagnosed AP (6.4%), acute cholecystitis (6.3%), and colon diverticulitis without perforation (5.2%) in the group with hospitalization for examination (Table S4). The diagnoses in >5% of patients were undiagnosed AP (23.7%), infectious enteritis (13.9%), ureterolithiasis (12.7%), and constipation (6.1%) in the group without hospitalization for examination (Table S4).

Table 4 summarizes the number and percentage of patients in the undiagnosed AP group with clinical findings corresponding to the AHP definition. All patients in the undiagnosed group had onset after adolescence and complained of gastrointestinal symptoms; 7.9% reported neurological or psychiatric symptoms, and 48.6% had autonomic symptoms. Critical examination, such as urinalysis for ALA or PBG, was performed in 0% of the patients despite 62.5% undergoing urinalysis (Table 4).

## Discussion

A certain percentage of patients are classified as having undiagnosed AP despite advanced diagnostic technology 2,8,19. This study revealed that 16.6% of the patients with AP who visited the general medicine departments of six JSHGM-accredited regional medical institutions in Japan had undiagnosed AP. Statistical analysis identified hospitalization for examination, dull pain (typically described as persistent, deep pain in an area, unlike sharp pain, and bearable pain during mild activities), and the absence of depressive symptoms as contributing factors to AP diagnosis. The urinary PBG test, which is the most crucial first-line screening test for diagnosing AHP 20, was not performed.

Hospitalization for examination was associated with AP diagnosis, likely because the diagnosed AP group included patients who required hospitalization for treatment, for example, those with acute appendicitis or bowel obstruction. ECG, abdominal ultrasonography, plain abdominal radiography, abdominal MRI, and blood gas examinations were more frequently performed in hospitalized patients than in non-hospitalized patients. Furthermore, image-specific diseases that became severe enough to require transvenous antimicrobial therapy or surgical treatment, for example, ileus or bowel obstruction, acute appendicitis, acute cholangitis, and acute cholecystitis, were more prevalent in patients who were hospitalized than in those who were not, based on the diagnoses of the diagnosed AP group. In contrast, undiagnosed AP, infectious enteritis, ureterolithiasis, and constipation were more prevalent among patients who were not hospitalized than among those who were hospitalized. These patients were likely to have mild or nonrecurrent AP that did not require close examination during hospitalization. The number of diagnosed cases is likely to increase over time through more detailed examinations. Considering a reasonable timeframe for diagnosis and detailed examinations in standard medical settings would be beneficial when establishing the criteria for NSAP, ensuring that all potential causes are captured, including any relevant rare diseases.

Dull pain in nontraumatic AP is more common in patients with severe disease than in those with mild disease 21, which may help diagnose AP requiring examination or treatment. Therefore, NSAP should be defined using factors that differentiate severe AP from mild AP to identify rare, diagnosable, and treatable conditions, as undiagnosed AP may include a certain percentage of mild disease. Conversely, depressive symptoms were associated with undiagnosed AP. Depression is associated with irritable bowel syndrome and functional AP 22-24. AP may be considered undiagnosed because of autonomic abnormalities, often associated with depression 24, or because depression is a somatoform disorder. Identifying the cause using imaging is difficult because these diseases are not organic. Similarly, AHP cannot be diagnosed using imaging because of its functional nature. A strategy for distinguishing rare diseases, such as AHP, mitochondrial disorders 25, and hereditary angioedema 26, due to NSAP is crucial after defining NSAP.

This study revealed that none of the patients with undiagnosed AP underwent a definitive diagnostic urinalysis for AHP. AHP is a rare but prognostically relevant and treatable disease associated with severe AP 20,27,28. Although its prevalence in Japan is unknown, it is estimated at 1 in 100,000 persons in Europe 13. Therefore, estimating the number of patients with AHP among the 317 undiagnosed patients with AP is challenging in this study. However, all 317 patients with undiagnosed AP had at least two clinical symptoms of AHP, including onset after adolescence and gastrointestinal symptoms, with approximately half of them having autonomic symptoms and 7.9% showing neurological or psychiatric symptoms. Nervous system and mood disorders are frequently associated with AHP 10,29, and in recent years, neurological symptoms combined with AP have been identified as key indicators of AHP 10,30. Although urinalysis was performed in 62.5% of the patients, urinary ALA or PBG levels, which are essential for diagnosing AHP, were not measured. The frequency of AHP in these patients could not be determined because AHP was not suspected or tested. The omission of diagnostic tests for rare diseases in NSAP evaluation results in a significant number of undiagnosed cases. In Japan, the number of diagnosed patients with AHP is markedly lower than the reported prevalence in Europe.

Testing for ALA and PBG was likely not performed for the following reasons: AHP is a rare disease; the symptoms vary and are uncharacteristic 31; a urine sample must be obtained during the attack phase 31,32. Identifying populations with a high prevalence of AHP and incorporating rare diseases into the diagnostic workup are crucial to ensure accurate diagnosis and appropriate management. Further studies are warranted to propose criteria for NSAP and determine AHP prevalence in NSAP. Comparing the characteristics of the abdominal pain population in our study with 391 cases of porphyria 10, our population was older (55 years vs. 44 years), had less abdominal pain throughout the abdomen (5.5% vs. 13.6%), more nausea (36.4% vs. 17.9%), and less insomnia (2.7% vs. 23.0%) and anxiety (1.3% vs. 11.3%). These differences in background characteristics would explain why AHP was not diagnosed in this study, and these characteristics would be crucial for diagnosing AHP among patients with abdominal pain. The test is unavailable in many facilities because of the rarity of the disease, and obtaining results takes 1-2 weeks 13. Both ALA and PBG levels are at least five times the upper limit of normal during acute AHP attack 13,33. The rapid qualitative PBG urine test has been available since the 1950s but has limited clinical use. However, a newly approved rapid test for PBG in the United States may improve diagnosis, as some patients experience delayed diagnoses of up to 15 years after symptom onset 13,31. Further studies on rapid tests, NSAP population identification, and appropriate implementation strategies for diagnostic tests could help distinguish AHP from undiagnosed AP.

This study has some limitations. First, this was a retrospective observational study, and factors not recorded in the medical files were treated as unknown, which may differ from the actual situation. Thus, prospective studies are needed to confirm the association between each factor and unknown diagnoses. Second, the number of participating facilities was small, and only hospitals, not clinics, were included. The characteristics of diagnosed and undiagnosed AP may differ among primary care settings. Third, the coronavirus disease pandemic coincided with the study period, which may have influenced care-seeking behavior. Patients may have refrained from seeking care for mild AP, and examinations requiring imaging may have been postponed, potentially leading to missed diagnoses. With the recent availability of medications for AHP 27, future survey updates will help to understand the clinical situation.

## Conclusions

Among patients with AP who visited the general medicine departments of six JSHGM-accredited regional medical institutions in Japan, 16.6% were classified as having undiagnosed AP. Diagnosed AP was associated with dull pain and hospitalization for examination, whereas undiagnosed AP was associated with the presence of depressive symptoms. None of the patients diagnosed with AP were also diagnosed with AHP, and only one patient was tested for AHP. Importantly, none of the patients with undiagnosed AP were tested for AHP, which is an underdiagnosed condition with chronic symptoms and potential long-term complications but with treatment/management options 10,26. Establishing a diagnostic strategy for undiagnosed AP would provide better opportunities for managing patients with NSAP and could help shorten the diagnostic journey for those with rare, difficult-to-diagnose diseases, such as AHP.

## Supplementary Material

Supplementary tables.

## Figures and Tables

**Table 1:** Baseline participant characteristics

		Undiagnosed (N=317)		Diagnosed (N=1598)		Odds ratio (95% CI)
		n		n		
Age	[years], median (Q1-Q3)	317	55 (39-72)	1598	57 (37-72.3)	1.00 (0.99-1.00)
Sex: male	n (%)		134 (42.3)		759 (47.5)	0.81 (0.63-1.03)
Body mass index	[kg/m^2^]					0.99 (0.96-1.03)
Male	median (Q1-Q3)	59	23.0 (20.7-24.4)	457	23.2 (20.5-25.6)	
Female	median (Q1-Q3)	93	22.0 (20.0-24.9)	503	22.5 (19.2-25.2)	
Visit						1.44 (1.13-1.83)*
Weekday daytime	n (%)		164 (51.7)		681 (42.6)	
Out-of-hours	n (%)		153 (48.3)		913 (57.1)	
History of being transported by ambulance	n (%)		82 (25.9)		530 (33.2)	1.43 (1.09-1.88)*
Number of visits due to abdominal pain	[times], median (Q1-Q3)		2 (1-3)		2 (1-5)	1.09 (1.05-1.13)**
No history of visiting other departments	n (%)		217 (68.5)		1162 (72.7)	0.90 (0.69-1.18)
History of being referred to the general medicine department	n (%)		87 (27.4)		395 (24.7)	0.87 (0.66-1.14)
Onset time of abdominal pain symptoms	[days ago], median (Q1-Q3)	281	2 (0.292-21)	1445	1 (0.208-4)	1.00 (1.00-1.00)*
Number of abdominal pain episodes between the onset of abdominal pain and the first visit	[times], median (Q1-Q3)	44	1 (1-1.3)	325	1 (1-1)	1.00 (0.96-1.03)

Univariate logistic analysis, *p<0.05, **p<0.001. Abbreviations: CI, confidence interval; Q1, first quartile; Q3, third quartile.

**Table 2:** Variables and odds ratios for the presence or absence of diagnosis with a *p*-value of <0.05 in the univariate logistic analysis

Explanatory variable	Odds ratio (95% CI)	*p*-value
Hospitalization for examination^1)^	4.52 (3.28-6.22)	<0.001
Blood test result: Mg^2)^	3.67 (2.21-6.08)	<0.001
Medication for pain: Opioid^1)^	2.96 (1.36-6.44)	0.006
Abdominal imaging: Abdominal plain radiography^2)^	2.10 (1.63-2.69)	<0.001
Abdominal imaging: Abdominal MRI^2)^	2.00 (1.12-3.59)	0.020
Blood test result: A/G ratio^2)^	1.90 (1.47-2.44)	<0.001
Blood test result: Plasma osmolality^2)^	1.72 (1.33-2.22)	<0.001
Characteristics of pain: Dull pain^1)^	1.68 (1.10-2.57)	0.016
Exacerbating factor: None^1)^	1.65 (1.21-2.25)	0.002
Medication for pain: Non-opioid^1)^	1.63 (1.27-2.11)	<0.001
Blood test result: Total protein^2)^	1.51 (1.05-2.18)	0.025
Blood test result: HBs antigen^2)^	1.46 (1.09-1.95)	0.010
Gastrointestinal disorder: Nausea or vomiting^1)^	1.45 (1.11-1.89)	0.006
Visit time: Weekday daytime	1.44 (1.13-1.83)	0.003
Blood test: Blood gas^2)^	1.44 (1.13-1.84)	0.003
Visit: History of being transported by ambulance^1)^	1.43 (1.09-1.88)	0.010
ECG^2)^	1.41 (1.10-1.80)	0.006
Blood test result: HCV antibody^2)^	1.40 (1.05-1.87)	0.020
Gastrointestinal disorder	1.36 (1.04-1.78)	0.025
Body temperature [°C]	1.31 (1.10-1.55)	0.002
Blood test result: Ca^2)^	1.30 (1.01-1.66)	0.038
No. of visits due to abdominal pain [times]	1.09 (1.05-1.13)	<0.001
Respiratory rate [breaths/min]	1.04 (1.01-1.08)	0.025
Pulse rate [beats/min]	1.01 (1.00-1.02)	0.031
Blood test result: Uric acid^2)^	0.68 (0.53-0.86)	0.020
Blood test result: Triglyceride^2)^	0.67 (0.53-0.86)	0.002
Blood test result: HDL-C^2)^	0.60 (0.46-0.77)	<0.001
Exacerbating factor: Food^1)^	0.60 (0.38-0.95)	0.028
Medication for pain^1)^	0.60 (0.46-0.78)	<0.001
Blood test result: LDL-C^2)^	0.59 (0.46-0.76)	<0.001
Characteristics of pain: Other^1)^	0.58 (0.42-0.79)	<0.001
Exacerbating factor: Other^1)^	0.43 (0.27-0.67)	<0.001
Comorbidity with psychiatric disorder: Anxiety^1)^	0.42 (0.18-0.95)	0.038
1) Presence (with) or absence (without), with absence (without) as the reference		
2) Examined or not examined, with not examined as the reference		

Abbreviations: CI, confidence interval; Mg, magnesium; MRI, magnetic resonance imaging; A/G ratio, albumin-globulin ratio; HBs antigen, hepatitis B surface antigen; ECG, electrocardiography; HCV, hepatitis C virus; Ca, calcium; HDL-C, high-density lipoprotein cholesterol; LDL-C, low-density lipoprotein cholesterol.

**Table 3:** Variables and odds ratios for the presence or absence of diagnosis with a *p*-value of <0.05 in the multivariate logistic analysis

Explanatory variable	Odds ratio (95% CI)	*p*-value
Hospitalization for examination	5.07 (2.11-12.20)	<0.001
Characteristics of pain: dull pain	4.47 (1.30-15.40)	0.017
Comorbidity with psychiatric disorder: depression	0.13 (0.02-0.73)	0.021

Odds ratios >1 and <1 represent the association of the parameters with diagnosed and undiagnosed abdominal pain, respectively. Multiple logistic analysis was conducted using the following explanatory variables: age; sex; visit; history of being transported by ambulance; onset time of abdominal pain symptoms (days before); electrocardiography; blood tests conducted; urinalysis; characteristics of pain: sharp pain; characteristics of pain: dull pain; characteristics of pain: colic pain; exacerbating factor: none; exacerbating factor: food; medication for pain: non-opioid; medication for pain: opioid; hospitalization for examination; gastrointestinal disorder: nausea or vomiting; neurological disorders; psychiatric disorders: anxiety; psychiatric disorders: insomnia; psychiatric disorders: depression; body temperature; blood pressure: systolic blood pressure; pulse rate; and respiratory rate. Abbreviation: CI, confidence interval

**Table 4:** Diagnosis status for acute hepatic porphyria in patients with undiagnosed abdominal pain

	Undiagnosed (N=317)	
	n	%
Clinical finding		
Onset after adolescence	317	100.0
With gastrointestinal symptoms	317	100.0
With neurological or psychiatric symptoms	25	7.9
With autonomic symptoms	154	48.6
With skin symptoms	0	0.0
Reference information for AHP diagnosis		
With family history	2	0.6
Drugs exacerbate abdominal pain	1	0.3
Menstruation exacerbates abdominal pain	0	0.0
With metabolic and nutritional disorders	17	5.4
Laboratory finding		
Urinalysis conducted	198	62.5
ALA measurement	0	0.0
PBG measurement	0	0.0

Abbreviations: AHP, acute hepatic porphyria; ALA, 5-aminolevulinic acid; PBG, porphobilinogen.

## Abbreviations

AP: abdominal pain
NSAP: non-specific abdominal pain
CT: computed tomography
AHP: acute hepatic porphyria
ALA: aminolevulinic acid
PBG: porphobilinogen
JSHGM: Japanese Society of Hospital General Medicine
