# Supplementary material for: Features of Undiagnosed Abdominal Pain and Diagnostic Status of Acute Hepatic Porphyria in Japan: A Retrospective Study
Source: Int J Med Sci. 2025 Jun 20;22(12):3014–21. doi: 10.7150/ijms.107826 (PMC12244078; doi:10.7150/ijms.107826)
Supplement: Supplementary file 1 — Supplementary tables. [file ijmsv22p3014s1.pdf]

Supplementary Tables

Table S1. List of medical institutions that participated in the study

Table S2. Explanatory variables in univariate logistic regression analysis

Table S3. Summary of diagnostic criteria for acute hepatic porphyria

Table S4. Breakdown of final diagnosis and number of patients with and without hospitalization for examination

Table S5. Association of abdominal imaging examinations with diagnosis of abdominal pain

Table S6. Association of examination status (With vs. Without examination) with diagnosis of abdominal pain

Table S7. Association of abdominal pain symptoms (location, characteristics, mode, exacerbating factor, and medication) with diagnosis of abdominal pain

Table S8. Association of associated findings other than abdominal pain with diagnosis of abdominal pain

Table S9. Association of other characteristics with diagnosis of abdominal pain

Table S10. Explanatory variables used in multivariate logistic analysis

Table S11. Detailed examinations in patients with undiagnosed or diagnosed abdominal pain with and without hospitalization for examination

Table S1. List of medical institutions that participated in the study

Institution	Department
Saga University Hospital	Department of General Medicine
JA Onomichi General Hospital	Department of Critical Care and General Medicine
Toho University School of Medicine	Department of General Medicine and Emergency Care
Fukuoka University Hospital	General Medicine
Juntendo University Faculty of Medicine	Department of General Medicine
Dokkyo Medical University	Department of Diagnostic and Generalist Medicine

Table S2. Explanatory variables in univariate logistic regression analysis

Parameter	Variable
Age	Continuous
Sex	Male*, Female
Height	Continuous
Body weight	Continuous
BMI	Continuous
Visit	Weekday daytime*, Out of hours
History of being transported by ambulance	No*, Yes
Number of visits due to abdominal pain	Continuous
History of being referred to the general medicine department	No*, Yes
History of visiting other departments	No*, Yes
Onset time of abdominal pain symptoms (days before)	Continuous
Number of abdominal pains between the onset of abdominal pain and the first visit	Continuous
ECG	No*, Yes
Blood tests conducted	No*, Yes
Blood test, each examination	Without check*, With check
Urinalysis	No*, Yes
Abdominal Imaging conducted	No*, Yes
Abdominal Imaging, each examination	Without check*, With check
Transvaginal ultrasound (female only)	No*, Yes
Location of pain	Whole*, Epigastrium (central upper abdomen), Right upper abdomen, Left upper abdomen, Central middle abdomen, Right middle abdomen, Left middle abdomen, Central lower abdomen, Right lower abdomen, Left lower abdomen
Characteristics of pain, each pain	Without check*, With check
Pain intervals, each interval	Without check*, With check
Mode of onset	Sudden*, Non-sudden
Exacerbating factor, each factor	Without check*, With check
Medication for pain, each medication	Without check*, With check
Hospitalization for examination	No*, Yes
Gastrointestinal disorders	No*, Yes
Gastrointestinal disorders, detail	Without check*, With check
Neurological disorders	No*, Yes
Neurological disorders, detail	Without check*, With check
Psychiatric disorders	No*, Yes
Psychiatric disorders, detail	Without check*, With check
Photosensitivity	No*, Yes
Photosensitivity, detail	Without check*, With check
Body temperature	Continuous
Systolic blood pressure	Continuous
Diastolic blood pressure	Continuous
Pulse rate	Continuous
Respiratory rate	Continuous
SpO_2	Continuous
Na value at first visit	Continuous
Family history of abdominal pain	No*, Yes
Urinalysis, ALA	No*, Yes
Urinalysis, ALA result	Continuous
Urinalysis, ALA test frequency	Continuous
Urinalysis, PBG	No*, Yes
Urinalysis, PBG result	Continuous
Urinalysis, PBG test frequency	Continuous
Survival status	Survival*, Death

*: Reference

Abbreviations: BMI, body mass index; ECG, electrocardiogram; SpO_2, saturation of percutaneous oxygen; ALA, 5-aminolevulinic acid; PBG, porphobilinogen

Table S3. Summary of diagnostic criteria for acute hepatic porphyria[1)]

Acute hepatic porphyria is defined as any of the following:
• Abnormalities in any of the clinical findings and urinary findings (both ALA and PBG) and for other diseases that rule out a diagnosis of exclusion.
• Any clinical findings and genetic abnormalities that rule out the diagnosis of exclusion.

Clinical findings	
1	Onset after adolescence. Onset is often acute
2	Various degrees of abdominal pain, vomiting, constipation (gastrointestinal symptoms)
3	Weakness of the limbs, convulsions, mental abnormalities (neuropsychiatric symptoms)
4	Hypertension, tachycardia, pyrexia, etc. (autonomic symptoms)
5	Cutaneous symptoms (photosensitivity)
Diagnostic references	
6	With family history
7	Abdominal pain is exacerbated by medication.
8	Abdominal pain is exacerbated with menstruation.
9	Associated with metabolic and nutritional disorders.
Diagnosis of exclusion	
1	Acute abdomen based on organic lesions (e.g. gallstone attacks, calculus urinary)
2	Ileus
3	Appendicitis
4	Dissociative disorders
5	Lead poisoning
6	Other porphyria

* Defined based on the clinical findings of the diagnostic criteria for acute porphyria as described in the Intractable Disease Information Centre's Porphyria “Overview, Diagnostic Criteria, etc.”
1) Summarized by authors referencing the website of Japan Intractable Disease Information Centre, “Porphyria (Designated Intractable Disease 254)” https://www.nanbyou.or.jp/entry/5546 (accessed 16/7/2023)

Table S4. Breakdown of final diagnosis and number of patients with and without hospitalization for examination

	Overall		Hospitalization for examination			
			With		Without	
Final diagnosis	N	%	n	%	n	%
Infectious enteritis	221	11.5	64	8.3	157	13.9
Ureterolithiasis	162	8.5	18	2.3	144	12.7
Acute appendicitis	114	6.0	77	10.0	37	3.3
Ileus or bowel obstruction (excluding associated with malignancy)	107	5.6	79	10.3	27	2.4
Constipation	83	4.3	14	1.8	69	6.1
Colon diverticulitis without perforation	69	3.6	40	5.2	26	2.3
Acute cholangitis	62	3.1	52	6.8	8	0.7
Acute cholecystitis	59	3.1	48	6.3	11	1.0
Ischemic enteritis	47	2.5	30	3.9	16	1.4
Irritable bowel syndrome	45	2.3	5	0.7	40	3.5
Benign mucosal lesions of the upper gastrointestinal tract	42	2.2	17	2.2	23	2.0
Acute pancreatitis	35	1.8	26	3.4	8	0.7
Extrinsic factors	31	1.6	12	1.6	18	1.6
Lower gastrointestinal perforation	30	1.6	22	2.9	8	0.7
Gallstone attack	30	1.6	9	1.2	21	1.9
Benign mucosal lesions of the esophagus	24	1.3	1	0.1	23	2.0
Dysmenorrhea	23	1.2	0	0.0	22	1.9
Pyelonephritis	22	1.1	13	1.7	8	0.7
Functional dyspepsia	20	1.0	2	0.3	18	1.6
Others (<20 cases）	374	19.5	183	23.8	179	15.8
No diagnosis	317	16.6	49	6.4	268	23.7

Table S5. Association of abdominal imaging examinations with diagnosis of abdominal pain

	Overall N = 1915 n (%)	Undiagnosed N = 317 n (%)	Diagnosed N = 1598 n (%)	Odds ratio (95% CI)
Abdominal Imaging	1911 (99.8)	317 (100.0)	1594 (99.7)	
Abdominal ultrasound	624 (32.6)	97 (30.6)	527 (33.0)	1.12 (0.86–1.45)
Upper endoscopy	301 (15.7)	50 (15.8)	251 (15.7)	1.00 (0.72–1.39)
Lower endoscopy	252 (13.2)	43 (13.6)	209 (13.1)	0.96 (0.67–1.36)
Plain abdominal X-ray	965 (50.4)	112 (35.3)	853 (53.4)	2.10 (1.62–2.69)**
Abdominal CT scan	1801 (94.0)	294 (92.7)	1507 (94.3)	1.30 (0.81–2.08)
Abdominal MRI scan	139 (7.3)	13 (4.1)	126 (7.9)	2.00 (1.12–3.59)*

Univariate logistic analysis, *p <0.05, **p <0.001

Abbreviations: CI, confidence interval; CT, computed tomography; MRI, magnetic resonance imaging

Table S6. Association of examination status (With vs Without examination) with diagnosis of abdominal pain

	Undiagnosed N = 317 n (%)	Diagnosed N = 1598 n (%)	Odds ratio (95% CI)
ECG	140 (44.2)	848 (53.1)	1.41 (1.10–1.80)*
Blood test	303 (95.6)	1536 (96.1)	1.14 (0.63–2.07)
Liver			
AST	267 (84.2)	1352 (84.6)	1.03 (0.74–1.43)
ALT	301 (95.0)	1529 (95.7)	1.18 (0.67–2.06)
γ-GT	300 (94.6)	1520 (95.1)	1.10 (0.64–1.89)
ALP	283 (89.3)	1473 (92.2)	1.42 (0.95–2.11)
Total bilirubin	290 (91.5)	1494 (93.5)	1.34 (0.86–2.08)
LDH	298 (94.0)	1514 (94.7)	1.15 (0.69–1.92)
Total protein	274 (86.4)	1448 (90.6)	1.51 (1.05–2.18)*
Albumin	282 (89.0)	1442 (90.2)	1.15 (0.78–1.69)
A/G ratio	110 (34.7)	802 (50.2)	1.90 (1.47–2.44)**
ChE	139 (43.8)	783 (49.0)	1.23 (0.97–1.57)
Hepatitis			
HBs antigen	68 (21.5)	456 (28.5)	1.46 (1.09–1.95)*
HBs antibody	34 (10.7)	224 (14.0)	1.36 (0.93–1.99)
HCV antibody	70 (22.1)	455 (28.5)	1.40 (1.05–1.87)*
Lipids			
Triglyceride	139 (43.8)	551 (34.5)	0.67 (0.53–0.86)*
HDL-C	118 (37.2)	418 (26.2)	0.60 (0.46–0.77)**
LDL-C	120 (37.9)	424 (26.5)	0.59 (0.46–0.76)**
Carbohydrate metabolism			
Blood glucose	243 (76.7)	1280 (80.1)	1.23 (0.92–1.63)
HbA1c	122 (38.5)	537 (33.6)	0.81 (0.63–1.04)
Uric acid, inflammation, kidney, pancreas			
Uric acid	180 (56.8)	752 (47.1)	0.68 (0.53–0.86)*
CRP	296 (93.4)	1513 (94.7)	1.26 (0.77–2.07)
BUN	297 (93.7)	1520 (95.1)	1.31 (0.79–2.18)
Creatinine	298 (94.0)	1519 (95.1)	1.23 (0.73–2.05)
e-GFR	295 (93.1)	1498 (93.7)	1.12 (0.69–1.80)
Amylase	254 (80.1)	1318 (82.5)	1.17 (0.86–1.58)
Blood cell			
WBC count	301 (95.0)	1528 (95.6)	1.16 (0.67–2.02)
RBC count	301 (95.0)	1528 (95.6)	1.16 (0.67–2.02)
Hemoglobin	301 (95.0)	1527 (95.6)	1.14 (0.66–1.99)
Hematocrit	301 (95.0)	1528 (95.6)	1.16 (0.67–2.02)
MCV	301 (95.0)	1527 (95.6)	1.14 (0.66–1.99)
MCH	301 (95.0)	1527 (95.6)	1.14 (0.66–1.99)
MCHC	301 (95.0)	1527 (95.6)	1.14 (0.66–1.99)
Platelet count	301 (95.0)	1526 (95.5)	1.13 (0.65–1.96)
WBC differential	294 (92.7)	1502 (94.0)	1.22 (0.76–1.96)
Electrolytes			
Na, K, Cl	299 (94.3)	1516 (94.9)	1.11 (0.66–1.88)
Ca	184 (58.0)	1026 (64.2)	1.30 (1.01–1.66)*
Mg	17 (5.4)	275 (17.2)	3.67 (2.21–6.08)**
Tumor marker			
AFP	3 (0.9)	28 (1.8)	1.87 (0.56–6.18)
CEA	35 (11.0)	219 (13.7)	1.28 (0.88–1.87)
PSA	3 (0.9)	24 (1.5)	1.60 (0.48–5.33)
CA125	12 (3.8)	38 (2.4)	0.62 (0.32–1.20)
CA15-3	2 (0.6)	5 (0.3)	0.49 (0.10–2.56)
Elastase	0 (0)	9 (0.6)	NA
CYFRA 21-1	0 (0)	11 (0.7)	NA
CA19-9	31 (9.8)	206 (12.9)	1.37 (0.92–2.03)
Blood gas	138 (43.5)	842 (52.7)	1.44 (1.13–2.22)*
Other			
Homocysteine	0 (0)	2 (0.1)	NA
Hepatitis	100 (31.5)	706 (44.2)	1.72 (1.33–2.22)**
Urinalysis	198 (62.5)	905 (56.6)	0.78 (0.61–1.00)*
ALA	0 (0)	1 (0.1)	NA
PBG	0 (0)	2 (0.1)	NA
Transvaginal ultrasound (female only)	7 (4.0)	52 (6.8)	1.69 (0.76–3.78)
Hospitalization for examination	49 (15.5)	719 (45.0)	4.52 (3.28–6.22)**

Univariate logistic analysis, *p <0.05, **p <0.001; NA: Not applicable

Abbreviations: ECG, electrocardiogram; AST, aspartate aminotransferase; ALT, alanine aminotransferase; γ-GT, gamma-glutamyltransferase; ALP, alkaline phosphatase; LDH, lactate dehydrogenase; A/G ratio, albumin/globulin ratio; ChE, cholinesterase; HBs antigen, hepatitis B surface antigen; HBs antibody, hepatitis B surface antibody; HCV antibody, hepatitis C virus antibody; HDL-C, high-density lipoprotein cholesterol; LDL-C, low-density lipoprotein cholesterol; HbA1c, hemoglobin A1c; CRP, C-reactive protein; BUN, blood urea nitrogen; e-GFR, estimated glomerular filtration rate; WBC, white blood cell; RBC, red blood cell; MCV, mean corpuscular volume; MCH, mean corpuscular hemoglobin; MCHC, mean corpuscular hemoglobin concentration; Na, Sodium; K, potassium; Cl, chlorine; Ca, calcium; Mg, magnesium; AFP, alpha-fetoprotein; CEA, carcinoembryonic antigen; PSA, prostate-specific antigen; CA125, cancer antigen 125; CA15-3, cancer antigen 15-3; CYFRA 21-1, cytokeratin fragment 21-1; CA19-9, cancer antigen 19-9; ALA, 5-aminolevulinic acid; PBG, porphobilinogen

Table S7. Association of abdominal pain symptoms (location, characteristics, mode, exacerbating factor, and medication) with diagnosis of abdominal pain

	**Undiagnosed (N = 317)** n (%)	**Diagnosed (N = 1598)** n (%)	**Odds ratio (95% CI)**
Location of pain			
Whole	20 (6.3)	86 (5.4)	
Central region of upper abdomen)	88 (27.8)	344 (21.5)	0.91 (0.53–1.56)
Right upper quadrant	17 (5.4)	98 (6.1)	1.34 (0.66–2.72)
Left upper quadrant	14 (4.4)	29 (1.8)	0.48 (0.22–1.07)
Central region of abdomen	8 (2.5)	78 (4.9)	2.27 (0.95–5.44)
Right side of the abdomen	22 (6.9)	99 (6.2)	1.05 (0.54–2.05)
Left side of the abdomen	12 (3.8)	105 (6.6)	2.03 (0.94–4.40)
Central region of lower abdomen	35 (11.0)	231 (14.5)	1.53 (0.84–2.80)
Right lower quadrant	41 (12.9)	241 (15.1)	1.37 (0.76–2.46)
Left lower quadrant	26 (8.2)	118 (7.4)	1.06 (0.55–2.01)
Characteristics of pain			
Sharp pain	54 (17.0)	250 (15.6)	1.25 (0.90–1.76)
Dull pain	29 (9.1)	176 (11.0)	1.68 (1.10–2.57)*
Colic pain	3 (0.9)	28 (1.8)	2.43 (0.73–8.06)
Peristaltic pain	6 (1.9)	20 (1.3)	0.85 (0.34–2.14)
Other pain	169 (53.3)	550 (34.4)	0.58 (0.42–0.79)**
Mode of onset			
Sudden	95 (30.0)	449 (28.1)	0.98 (0.74–1.29)
Non-sudden	162 (51.1)	749 (46.9)	
Exacerbating factor			
None	131 (41.3)	899 (56.3)	1.65 (1.21–2.25)*
Food	26 (8.2)	96 (6.0)	0.60 (0.38–0.95)*
Body movement	20 (6.3)	126 (7.9)	1.08 (0.66–1.77)
Posture	19 (6.0)	82 (5.1)	0.72 (0.42–1.21)
Menstruation	0 (0.0)	0 (0.0)	—
Other	29 (9.1)	79 (4.9)	0.43 (0.27–0.67)**
Medication for pain			
None	105 (33.1)	385 (24.1)	0.60 (0.46–0.78)**
Non-opioid analgesics	158 (49.8)	1007 (63.0)	1.63 (1.27–2.11)**
Opioid analgesics	7 (2.2)	104 (6.5)	2.96 (1.36–6.44)**
Analgesic adjuvant	11 (3.5)	49 (3.1)	0.84 (0.43–1.64)
Other	56 (17.7)	232 (14.5)	0.75 (0.54–1.04)

Univariate logistic analysis, *p <0.05, **p <0.001
Abbreviation: CI, confidence interval

Table S8. Association of associated findings other than abdominal pain with diagnosis of abdominal pain

	**Undiagnosed (N = 317)** n (%)	**Diagnosed (N = 1598)** n (%)	**Odds ratio (95% CI)**
No gastrointestinal disorder	102 (33.2)	399 (25.0)	
With gastrointestinal disorder	181 (57.1)	964 (60.3)	1.36 (1.04–1.78)*
Nausea/vomiting	99 (31.2)	597 (37.4)	1.45 (1.11–1.89)*
Constipation	46 (14.5)	175 (11.0)	0.76 (0.53–1.08)
Diarrhea	69 (21.8)	345 (21.6)	1.05 (0.78–1.42)
Other	31 (9.8)	178 (11.1)	1.22 (0.81–1.83)
No neurological disorder	235 (74.1)	931 (58.3)	
With neurological disorder	20 (6.3)	88 (5.5)	1.11 (0.67–1.84)
Numbness/pain in extremities	9 (2.8)	30 (1.9)	0.83 (0.39–1.77)
Weakness in extremities	1 (0.3)	9 (0.6)	2.26 (0.29–17.90)
Convulsion	1 (0.3)	4 (0.3)	1.00 (0.11–8.99)
Other	12 (3.8)	58 (3.6)	1.22 (0.65–2.31)
No psychiatric disorder	225 (71.0)	918 (57.4)	
With psychiatric disorder	37 (11.7)	178 (11.1)	1.18 (0.80–1.73)
Anxiety	9 (2.8)	16 (1.0)	0.42 (0.18–0.95)*
Insomnia	13 (4.1)	38 (2.4)	0.69 (0.36–1.31)
Depression	6 (1.9)	23 (1.4)	0.92 (0.37–2.27)
Disorientation	0 (0.0)	1 (0.1)	—
Other	24 (7.6)	133 (8.3)	1.37 (0.87–2.16)
No photosensitivity	115 (36.3)	575 (36.0)	
With photosensitivity	0 (0.0)	0 (0.0)	—
Rash	0 (0.0)	0 (0.0)	
Blister	0 (0.0)	0 (0.0)	

Univariate logistic analysis, *p <0.05
Abbreviation: CI, confidence interval

Table S9. Association of other characteristics with diagnosis of abdominal pain

Vital signs at initial visit		Undiagnosed (N = 317) n	Undiagnosed (N = 317) Median (Q1–Q3)	Diagnosed (N = 1598) n	Diagnosed (N = 1598) Median (Q1–Q3)	Odds ratio (95% CI)
Body temperature	°C	293	36.6 (36.3–36.9)	1516	36.7 (36.3–37.1)	1.31 (1.10–1.55)*
Systolic blood pressure	mmHg	293	135 (120–152)	1504	130 (114–149)	0.99 (0.99–1.00)*
Diastolic blood pressure	mmHg	292	81 (79–92)	1500	80 (69–90)	0.99 (0.99–1.00)*
Pulse rate	beats/min	290	80 (70.3–90.8)	1483	83 (72–95)	1.01 (1.00–1.02)*
Respiratory rate	breaths/min	191	16 (14–20)	1059	18 (16–20)	1.04 (1.01–1.08)*
SpO_2	%	273	98 (97–98)	1408	98 (97–99)	1.02 (0.94–1.10)
Na value at first visit	mEq/L	282	140 (138–142)	1465	140 (138–142)	1.01 (0.99–1.04)

Family history of abdominal pain	Undiagnosed n (%)	Diagnosed n (%)	Odds ratio (95% CI)
With	2 (0.6)	2 (0.1)	0.22 (0.03–1.57)
No	149 (47.0)	681 (42.6)	
**Survival status**			
Survival	246 (77.6)	1139 (71.3)	2.07 (0.94–4.56)
Death	7 (2.2)	67 (4.2)	

Univariate logistic analysis, *$p < 0.05$

Abbreviations: CI, confidence interval; SpO_2, saturation of percutaneous oxygen

Table S10. Explanatory variables used in multivariate logistic analysis

Explanatory variable	Variable
Age	Continuous
Sex	Male*, Female
Visit: History of being transported by ambulance	No*, Yes
Onset time of abdominal pain symptoms (days before)	Continuous
ECG	No*, Yes
Blood tests conducted	No*, Yes
Urinalysis	No*, Yes
Characteristics of pain: Sharp pain	Without check*, With check
Characteristics of pain: Dull pain	Without check*, With check
Characteristics of pain: Colic pain	Without check*, With check
Exacerbating factor: None	Without check*, With check
Exacerbating factor: Food	Without check*, With check
Medication for pain: Non-opioid	Without check*, With check
Medication for pain: Opioid	Without check*, With check
Hospitalization for examination	No*, Yes
Gastrointestinal disorder: Nausea or vomiting	Without check*, With check
Neurological disorders	No*, Yes
Psychiatric disorders: Anxiety	Without check*, With check
Psychiatric disorders: Insomnia	Without check*, With check
Psychiatric disorders: Depression	Without check*, With check
Body temperature	Continuous
Blood pressure: SBP	Continuous
Pulse rate	Continuous
Respiratory rate	Continuous

*: Reference

Abbreviations: ECG, electrocardiogram; SBP, systolic blood pressure

Table S11. Detailed examinations in patients with undiagnosed or diagnosed abdominal pain with and without hospitalization for examination

	Overall				Undiagnosed AP				Diagnosed AP			
	Without1)		With2)		Without1)		With2)		Without1)		With2)	
	n	%	n	%	n	%	n	%	n	%	n	%
ECG	382	33.8	599	78.0	100	37.6	40	81.6	282	32.6	559	77.7
Urinalysis	582	51.5	510	66.4	162	60.9	35	71.4	420	48.6	475	66.1
Abdominal Imaging	1126	99.6	768	100.0	266	100.0	49	100.0	860	99.5	719	100.0
Abdominal ultrasound	315	27.9	306	39.8	76	28.6	21	42.9	239	27.7	285	39.6
Upper endoscopy	144	12.7	153	19.9	36	13.5	13	26.5	108	12.5	140	19.5
Lower endoscopy	115	10.2	135	17.6	33	12.4	9	18.4	82	9.5	126	17.5
Plain abdominal X-ray	406	35.9	552	71.9	88	33.1	23	46.9	318	36.8	529	73.6
Abdominal CT scan	1025	90.7	761	99.1	243	91.4	49	100.0	782	90.5	712	99.0
Abdominal MRI scan	43	3.8	95	12.4	8	3.0	5	10.2	35	4.1	90	12.5
Transvaginal ultrasound (female only)	31	2.7	27	3.5	6	2.3	1	2.0	25	2.9	26	3.6
Blood test	1058	93.6	765	99.6	253	95.1	49	100.0	805	93.2	716	99.6
Blood gas	432	38.2	542	70.6	102	38.3	36	73.5	330	38.2	506	70.4

1) Without hospitalization for examination
2) With hospitalization for examination
Abbreviations: AP, abdominal pain; ECG, electrocardiogram; CT, computed tomography; MRI, magnetic resonance imaging
